# Neuropsychological functions as endophenotype markers in ocd: a long term follow-up

**DOI:** 10.1192/j.eurpsy.2024.1308

**Published:** 2024-08-27

**Authors:** M. Puialto, J. Segalas, M. D. P. Alonso, E. Real

**Affiliations:** Hospital Universitario de Bellvitge, L’Hospitalet de Llobregat, Spain

## Abstract

**Introduction:**

Obssesive Compulsive Disorder (OCD) is characterized by impaired neuropsychological functions that are also influenced by clinical variables and aging.

According to the literature, several of these neuropsychological deficits could be potential endophenotype markers.

**Objectives:**

The present study aimed to study what kind of cognitive deficits OCD patients have and how aging and clinical course modify their cognitive profiles campared with general population.

**Methods:**

This study examined a sample of 60 adult outpatients with OCD diagnosis, who were matched with 70 healthy controls (HC). Cognitive performance in both groups was assessed using a neuropsychological battery including Rey-Osterrieth complex Figure (ROCF) and Digit Span Test (DGS). Based on previous research on neuropsychology of OCD, it was specified that these neuropsychological measures could be divided in two composites. The first composite, Executive function, includes Total Digit Span and the domain of organization of ROCF. The second composite, Non-Verbal Memory, includes the copy of ROCF, immediate recall, delayed recall and recognition of ROCF.

Severity of OCD symptoms was assessed by YBOCS and HDRS was used for symptoms of depression.

Both cognitive performance and clinical data were documented before and after a follow-up of 11 years.

During analysis, group differences between patients with OCD and HC regarding demographic and clinical characteristics at baseline and follow-up were calculated with independent t-tests and Pearson tests.

The main analysis tested if the change in cognitive function over time differed between patients and controls. To this end, a linear mixed model was used, examining the interaction between age, gender and time in both groups.

**Results:**

Older age, in patients with OCD and HC, was associated with poorer performance on executive function and nonverbal memory. Executive function was influenced by severity of OCD, and non-verbal memory by depressive symptoms at baseline. While, after the follow-up, as obsessive and affective symptoms improve along de follow-up, there is no significant change in the neuropsychological pattern.

At baseline, patients with OCD showed a poorer performance than HC in areas of nonverbal memory and executive function. After de follow-up, there is a poorer performance in the cognitive function in both groups, as they get older. However, there is no significant difference in this change between patients and HC.

**Conclusions:**

Results suggest that OCD is characterized by the existence of dysfunction in several neuropsychological areas that are influenced by time and clinical variables.

Nevertheless, this alteration is no solely attributable to these factors, as they remain stable through time compared to the general population. Therefore, certain neuropsychological functions might be endophenotype traits of the disorder.

**Disclosure of Interest:**

None Declared

